# Supplementary material for: Multi-omics analyses reveal rumen microbes and secondary metabolites that are unique to livestock species
Source: mSystems. 2024 Jan 31;9(2):e01228-23. doi: 10.1128/msystems.01228-23 (PMC10878066; doi:10.1128/msystems.01228-23)
Supplement: Supplemental material — Supplemental figure and tables. [file msystems.01228-23-s0001.docx]

**Supplemental Information**

Applying multi-omics to understand the role of livestock rumen microbes and diet to secondary metabolite diversity and functionality in four livestock species

Victor O. Omondi^1, 2^, Geoffrey O. Bosire^2^, John M. Onyari^2^, Caleb Kibet^1^, Samuel Mwasya^1^, Natasha V. Onyonyi^1^, and Merid N. Getahun^1*^

^1^International Centre of Insect Physiology and Ecology (*icipe*), Kenya.

^2^Department of Chemistry, University of Nairobi (U.o.N), Kenya.

Corresponding author: Merid N. Getahun

**Variability in metabolite composition among individual species population**

The correlation between populations of the same species dynamics and the metabolite compound profiles of four types of livestock was investigated using Pearson’s correlation analysis (Fig. S1 A-D). Generally, a minimal variability in metabolites was observed between individuals of the same species. Cattle, goats, and camels showed minimal variability in their volatile organic compound profiles (Fig. S1A-C); however, sheep populations showed some variation in sheep 7, 8, 9, and 10 (Fig. S1D). As a result, the rumen odor profiles in the herd populations of the four cattle species used in this investigation were comparable.


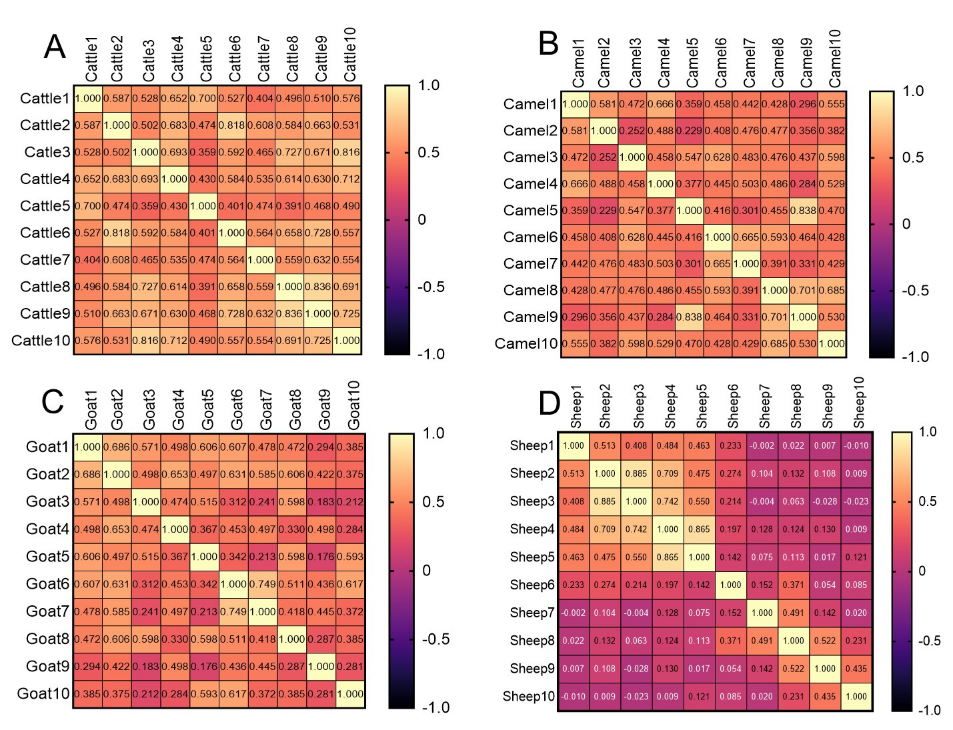


**Fig. S1**: Color coded matrix plots showing variability in metabolite compounds profiles within individual livestock species, (A) Cattle, (B) Camels, (C) Goat and (D) Sheep respectively. Analysis based on Pearson’s correlation of secondary metabolites relative abundance.

**Table S1**

Rumen bacteria abundance profile in livestock

|  | Genus | Cow | Camel | Goat | Sheep |
| --- | --- | --- | --- | --- | --- |
| 1 | *Rikenellaceae RC9 gut group* | 19.40% | 18.50% | 16.30% | 17.60% |
| 2 | *Christensenellaceae R-7 group* | 16.50% | 12.00% | 14.40% | 14.10% |
| 3 | *Prevotella* | 3.40% | 11.70% | 8.60% | 18.80% |
| 4 | *NK4A214 group* | 5.00% | 5.60% | 5.20% | 4.40% |
| 5 | *Saccharofermentans* | 7.10% | 0.90% | 4.30% | 3.70% |
| 6 | *Butyrivibrio* | 1.70% | 5.90% | 3.00% | 2.90% |
| 7 | *Psychrobacter* | 12.00% | 0.00% | 1.60% | 0.00% |
| 8 | *Lachnospiraceae XPB1014 group* | 2.20% | 0.90% | 2.90% | 4.00% |
| 9 | *Candidatus Saccharimonas* | 2.30% | 4.50% | 1.60% | 0.90% |
| 10 | *Succiniclasticum* | 2.30% | 1.50% | 3.00% | 1.90% |
| 11 | *Prevotellaceae UCG-003* | 0.30% | 4.30% | 1.40% | 2.20% |
| 12 | *Prevotellaceae UCG-001* | 0.70% | 4.00% | 1.30% | 1.00% |
| 13 | *[Ruminococcus] gauvreauii group* | 0.10% | 5.80% | 0.20% | 0.20% |
| 14 | *Lachnospiraceae AC2044 group* | 2.10% | 1.00% | 2.00% | 1.30% |
| 15 | *UCG-005* | 3.60% | 0.50% | 1.50% | 0.90% |
| 16 | *Prevotellaceae NK3B31 group* | 1.40% | 1.10% | 2.10% | 1.30% |
| 17 | *Lachnospiraceae NK3A20 group* | 0.40% | 3.00% | 1.50% | 0.60% |
| 18 | *Treponema* | 0.60% | 0.60% | 1.00% | 2.70% |
| 19 | *Family XIII AD3011 group* | 1.50% | 0.60% | 1.40% | 0.80% |
| 20 | *SP3-e08* | 0.30% | 0.20% | 2.20% | 1.30% |
| 21 | *Acinetobacter* | 0.00% | 0.00% | 2.00% | 1.80% |
| 22 | *p-1088-a5 gut group* | 1.40% | 0.60% | 0.80% | 0.40% |
| 23 | *Pseudobutyrivibrio* | 0.40% | 1.10% | 1.20% | 0.20% |
| 24 | *Anaerovorax* | 1.10% | 0.10% | 1.40% | 0.40% |
| 25 | *[Eubacterium] ruminantium group* | 1.20% | 0.20% | 0.60% | 1.00% |
| 26 | *Carnobacterium* | 2.20% | 0.00% | 0.80% | 0.20% |
| 27 | *Papillibacter* | 1.00% | 0.30% | 1.30% | 0.30% |
| 28 | *Shuttleworthia* | 0.20% | 0.90% | 0.80% | 0.50% |
| 29 | *Acetitomaculum* | 0.10% | 1.30% | 0.50% | 0.20% |
| 30 | *Ruminococcus* | 0.50% | 0.20% | 0.70% | 0.70% |
| 31 | *Lachnospiraceae UCG-008* | 0.50% | 0.40% | 0.70% | 0.40% |
| 32 | *Marvinbryantia* | 0.10% | 1.20% | 0.30% | 0.30% |
| 33 | *Prevotellaceae UCG-004* | 0.10% | 1.30% | 0.40% | 0.20% |
| 34 | *Lachnospiraceae UCG-006* | 0.80% | 0.30% | 0.70% | 0.20% |
| 35 | *Anaeroplasma* | 0.00% | 0.40% | 0.00% | 1.30% |
| 36 | *[Eubacterium] hallii group* | 0.40% | 0.70% | 0.30% | 0.30% |
| 37 | *Bacillus* | 0.00% | 0.00% | 0.00% | 1.40% |
| 38 | *Moryella* | 0.00% | 0.50% | 0.80% | 0.10% |
| 39 | *Pseudomonas* | 0.10% | 0.00% | 0.00% | 1.30% |
| 40 | *Lachnospiraceae NK4A136 group* | 0.20% | 0.00% | 0.80% | 0.40% |
| 41 | *probable genus 10* | 0.40% | 0.20% | 0.40% | 0.40% |
| 42 | *Blautia* | 0.10% | 0.80% | 0.30% | 0.20% |
| 43 | *Oribacterium* | 0.10% | 0.30% | 0.60% | 0.30% |
| 44 | *Desulfovibrio* | 0.20% | 0.10% | 0.60% | 0.20% |
| 45 | *Veillonellaceae UCG-001* | 0.20% | 0.10% | 0.60% | 0.10% |
| 46 | *Defluviitaleaceae UCG-011* | 0.10% | 0.10% | 0.60% | 0.20% |
| 47 | *Lachnospiraceae FCS020 group* | 0.20% | 0.10% | 0.30% | 0.40% |
| 48 | *Olsenella* | 0.10% | 0.50% | 0.10% | 0.10% |
| 49 | *Lachnospiraceae UCG-009* | 0.20% | 0.30% | 0.20% | 0.20% |
| 50 | *CPla-4 termite group* | 0.40% | 0.10% | 0.40% | 0.00% |
| 51 | *Ruminiclostridium* | 0.40% | 0.20% | 0.20% | 0.10% |
| 52 | *Lachnospiraceae ND3007 group* | 0.10% | 0.00% | 0.30% | 0.40% |
| 53 | *FD2005* | 0.00% | 0.70% | 0.00% | 0.10% |
| 54 | *Selenomonas* | 0.00% | 0.40% | 0.20% | 0.20% |
| 55 | *Family XIII UCG-001* | 0.10% | 0.40% | 0.10% | 0.10% |
| 56 | *Incertae Sedis* | 0.30% | 0.10% | 0.30% | 0.00% |
| 57 | *[Eubacterium] nodatum group* | 0.20% | 0.10% | 0.20% | 0.20% |
| 58 | *Lachnospira* | 0.00% | 0.30% | 0.20% | 0.20% |
| 59 | *Fretibacterium* | 0.10% | 0.00% | 0.10% | 0.40% |
| 60 | *UCG-002* | 0.20% | 0.00% | 0.20% | 0.30% |
| 61 | *U29-B03* | 0.10% | 0.20% | 0.20% | 0.10% |
| 62 | *V9D2013 group* | 0.20% | 0.00% | 0.20% | 0.10% |
| 63 | *Solobacterium* | 0.10% | 0.10% | 0.20% | 0.10% |
| 64 | *[Eubacterium] siraeum group* | 0.00% | 0.00% | 0.00% | 0.40% |
| 65 | *Roseburia* | 0.10% | 0.10% | 0.10% | 0.20% |
| 66 | *Pirellula* | 0.40% | 0.00% | 0.10% | 0.00% |
| 67 | *possible genus Sk018* | 0.00% | 0.30% | 0.10% | 0.10% |
| 68 | *Lachnospiraceae UCG-002* | 0.00% | 0.10% | 0.20% | 0.10% |
| 69 | *Syntrophococcus* | 0.10% | 0.00% | 0.20% | 0.20% |
| 70 | *Monoglobus* | 0.20% | 0.10% | 0.20% | 0.10% |
| 71 | *[Eubacterium] ventriosum group* | 0.30% | 0.00% | 0.10% | 0.10% |
| 72 | *UCG-007* | 0.20% | 0.00% | 0.20% | 0.10% |
| 73 | *Mogibacterium* | 0.10% | 0.10% | 0.20% | 0.10% |
| 74 | *Fibrobacter* | 0.10% | 0.10% | 0.00% | 0.10% |
| 75 | *Planomicrobium* | 0.10% | 0.00% | 0.30% | 0.00% |
| 76 | *UCG-004* | 0.00% | 0.20% | 0.00% | 0.10% |
| 77 | *Prevotellaceae Ga6A1 group* | 0.00% | 0.00% | 0.00% | 0.30% |
| 78 | *[Eubacterium] saphenum group* | 0.20% | 0.00% | 0.20% | 0.00% |
| 79 | *DNF00809* | 0.00% | 0.10% | 0.20% | 0.10% |
| 80 | *[Eubacterium] brachy group* | 0.10% | 0.00% | 0.10% | 0.10% |
| 81 | *Planococcus* | 0.30% | 0.00% | 0.00% | 0.00% |
| 82 | *Amnipila* | 0.10% | 0.00% | 0.10% | 0.10% |
| 83 | *Arthrobacter* | 0.00% | 0.00% | 0.10% | 0.10% |
| 84 | *Lachnospiraceae FE2018 group* | 0.00% | 0.10% | 0.10% | 0.10% |
| 85 | *Coprococcus* | 0.00% | 0.10% | 0.00% | 0.10% |
| 86 | *Streptococcus* | 0.00% | 0.10% | 0.00% | 0.10% |
| 87 | *[Eubacterium] xylanophilum group* | 0.00% | 0.00% | 0.00% | 0.10% |
| 88 | *Erysipelotrichaceae UCG-009* | 0.00% | 0.00% | 0.10% | 0.10% |
| 89 | *NED5E9* | 0.00% | 0.00% | 0.00% | 0.20% |
| 90 | *UCG-001* | 0.00% | 0.00% | 0.10% | 0.10% |
| 91 | *Lachnoclostridium* | 0.10% | 0.10% | 0.00% | 0.00% |
| 92 | *Schwartzia* | 0.10% | 0.00% | 0.00% | 0.00% |
| 93 | *Anaerovibrio* | 0.00% | 0.00% | 0.00% | 0.10% |
| 94 | *[Anaerorhabdus] furcosa group* | 0.00% | 0.00% | 0.10% | 0.00% |
| 95 | *Solibacillus* | 0.00% | 0.00% | 0.10% | 0.00% |
| 96 | *Atopobium* | 0.00% | 0.00% | 0.10% | 0.00% |
| 97 | *Desemzia* | 0.00% | 0.00% | 0.10% | 0.00% |
| 98 | *[Eubacterium] cellulosolvens group* | 0.00% | 0.00% | 0.00% | 0.10% |
| 99 | *Lachnospiraceae UCG-010* | 0.00% | 0.00% | 0.10% | 0.00% |
| 100 | *Agathobacter* | 0.00% | 0.10% | 0.00% | 0.00% |
| 101 | *Mailhella* | 0.00% | 0.00% | 0.00% | 0.00% |
| 102 | *Anaerofustis* | 0.10% | 0.00% | 0.00% | 0.00% |
| 103 | *UCG-009* | 0.00% | 0.00% | 0.10% | 0.00% |
| 104 | *DEV114* | 0.00% | 0.00% | 0.00% | 0.00% |
| 105 | *Denitrobacterium* | 0.00% | 0.10% | 0.00% | 0.00% |
| 106 | *Succinivibrio* | 0.00% | 0.10% | 0.00% | 0.00% |
| 107 | *Enterorhabdus* | 0.10% | 0.00% | 0.00% | 0.00% |
| 108 | *Alloprevotella* | 0.00% | 0.00% | 0.00% | 0.10% |
| 109 | *Sphaerochaeta* | 0.00% | 0.10% | 0.00% | 0.00% |
| 110 | *Lachnobacterium* | 0.00% | 0.00% | 0.00% | 0.10% |
| 111 | *Pseudoramibacter* | 0.00% | 0.00% | 0.00% | 0.00% |
| 112 | *[Eubacterium] oxidoreducens group* | 0.00% | 0.00% | 0.00% | 0.00% |
| 113 | *GCA-900066575* | 0.00% | 0.00% | 0.00% | 0.00% |
| 114 | *Paludicola* | 0.00% | 0.00% | 0.00% | 0.00% |
| 115 | *Catenisphaera* | 0.00% | 0.00% | 0.00% | 0.00% |
| 116 | *Quinella* | 0.00% | 0.00% | 0.00% | 0.00% |
| 117 | *Prevotellaceae YAB2003 group* | 0.00% | 0.00% | 0.00% | 0.10% |
| 118 | *Howardella* | 0.00% | 0.00% | 0.00% | 0.00% |
| 119 | *[Bacteroides] pectinophilus group* | 0.00% | 0.00% | 0.00% | 0.00% |
| 120 | *possible genus Sk003-Sk004* | 0.00% | 0.00% | 0.00% | 0.00% |
| 121 | *Sediminispirochaeta* | 0.00% | 0.00% | 0.00% | 0.00% |
| 122 | *Erysipelotrichaceae UCG-008* | 0.00% | 0.00% | 0.00% | 0.00% |
| 123 | *Prevotella_7* | 0.00% | 0.00% | 0.00% | 0.00% |
| 124 | *Anaerosporobacter* | 0.00% | 0.00% | 0.00% | 0.00% |
| 125 | *Tyzzerella* | 0.00% | 0.00% | 0.00% | 0.00% |
| 126 | *Catenibacillus* | 0.00% | 0.00% | 0.00% | 0.00% |
| 127 | *CAG-352* | 0.00% | 0.00% | 0.00% | 0.00% |
| 128 | *Pyramidobacter* | 0.00% | 0.00% | 0.00% | 0.00% |
| 129 | *Mycoplasma* | 0.00% | 0.00% | 0.00% | 0.00% |
| 130 | *Psychrobacillus* | 0.00% | 0.00% | 0.00% | 0.00% |
| 131 | *Desulfobulbus* | 0.00% | 0.00% | 0.00% | 0.00% |
| 132 | *Lachnospiraceae UCG-001* | 0.00% | 0.00% | 0.00% | 0.00% |
| 133 | *Anaerobiospirillum* | 0.00% | 0.00% | 0.00% | 0.00% |
| 134 | *Corynebacterium* | 0.00% | 0.00% | 0.00% | 0.00% |
| 135 | *Suttonella* | 0.00% | 0.00% | 0.00% | 0.00% |
| 136 | *Candidatus Soleaferrea* | 0.00% | 0.00% | 0.00% | 0.00% |
| 137 | *Succinivibrionaceae UCG-002* | 0.00% | 0.00% | 0.00% | 0.00% |
| 138 | *Shewanella* | 0.00% | 0.00% | 0.00% | 0.00% |
| 139 | *Raoultibacter* | 0.00% | 0.00% | 0.00% | 0.00% |
| 140 | *Sharpea* | 0.00% | 0.00% | 0.00% | 0.00% |
| 141 | *Erysipelotrichaceae UCG-006* | 0.00% | 0.00% | 0.00% | 0.00% |
| 142 | *Colidextribacter* | 0.00% | 0.00% | 0.00% | 0.00% |
|  | Total | 100.00% | 100.00% | 100.00% | 100.00% |

**S. table 2**

Rumen fungi abundance profiles in livestock

|  | Genus | Cow | Camel | Goat | Sheep |
| --- | --- | --- | --- | --- | --- |
| 1 | *Caecomyces* | 55.10% | 0.00% | 6.30% | 3.00% |
| 2 | *Neocallimastix* | 0.00% | 18.70% | 64.90% | 49.70% |
| 3 | *Anaeromyces* | 20.40% | 0.00% | 0.10% | 0.00% |
| 4 | *Pecoramyces* | 1.20% | 0.40% | 5.70% | 33.70% |
| 5 | *Cyllamyces* | 12.40% | 0.00% | 0.00% | 0.00% |
| 6 | *Orpinomyces* | 9.70% | 0.00% | 0.00% | 0.00% |
| 7 | *Cleistothelebolus* | 0.00% | 0.00% | 10.30% | 0.00% |
| 8 | *Oontomyces* | 0.00% | 53.40% | 0.00% | 0.00% |
| 9 | *Liebetanzomyces* | 0.20% | 0.00% | 0.20% | 7.00% |
| 10 | *Piromyces* | 0.20% | 13.20% | 1.30% | 3.20% |
| 11 | *Cladosporium* | 0.10% | 2.60% | 1.10% | 0.10% |
| 12 | *Nigrospora* | 0.00% | 0.20% | 1.00% | 0.80% |
| 13 | *Neodidymelliopsis* | 0.00% | 0.20% | 1.50% | 0.30% |
| 14 | *Ustilago* | 0.00% | 0.00% | 0.10% | 0.90% |
| 15 | *Aureobasidium* | 0.00% | 5.10% | 0.10% | 0.00% |
| 16 | *Aplosporella* | 0.00% | 0.30% | 0.70% | 0.00% |
| 17 | *Epicoccum* | 0.10% | 1.40% | 0.00% | 0.20% |
| 18 | *Naganishia* | 0.00% | 0.00% | 0.60% | 0.00% |
| 19 | *Curvularia* | 0.00% | 0.00% | 0.50% | 0.10% |
| 20 | *Sporormiella* | 0.00% | 0.20% | 0.50% | 0.10% |
| 21 | *Cystobasidium* | 0.00% | 0.00% | 0.40% | 0.00% |
| 22 | *Anthracocystis* | 0.10% | 0.00% | 0.00% | 0.20% |
| 23 | *Coniella* | 0.00% | 0.00% | 0.40% | 0.00% |
| 24 | *Gymnopus* | 0.00% | 0.00% | 0.30% | 0.00% |
| 25 | *Lyomyces* | 0.00% | 0.00% | 0.30% | 0.00% |
| 26 | *Hohenbuehelia* | 0.00% | 0.00% | 0.30% | 0.00% |
| 27 | *Neosulcatispora* | 0.00% | 0.20% | 0.20% | 0.00% |
| 28 | *Exserohilum* | 0.00% | 0.00% | 0.10% | 0.10% |
| 29 | *Filobasidium* | 0.00% | 1.50% | 0.00% | 0.00% |
| 30 | *Stagonosporopsis* | 0.10% | 0.00% | 0.00% | 0.00% |
| 31 | *Allophoma* | 0.00% | 0.00% | 0.20% | 0.00% |
| 32 | *Paraconiothyrium* | 0.00% | 0.00% | 0.20% | 0.00% |
| 33 | *Ectophoma* | 0.00% | 0.00% | 0.20% | 0.00% |
| 34 | *Arthrocladium* | 0.00% | 0.00% | 0.20% | 0.00% |
| 35 | *Paraboeremia* | 0.00% | 0.00% | 0.20% | 0.00% |
| 36 | *Podaxis* | 0.00% | 0.00% | 0.20% | 0.00% |
| 37 | *Hannaella* | 0.00% | 0.00% | 0.20% | 0.00% |
| 38 | *Hyphoderma* | 0.00% | 0.00% | 0.10% | 0.00% |
| 39 | *Neoascochyta* | 0.00% | 0.00% | 0.00% | 0.00% |
| 40 | *Cintractia* | 0.00% | 0.00% | 0.00% | 0.00% |
| 41 | *Symmetrospora* | 0.00% | 0.00% | 0.10% | 0.00% |
| 42 | *Wallemia* | 0.00% | 0.00% | 0.10% | 0.00% |
| 43 | *Alternaria* | 0.00% | 0.60% | 0.00% | 0.00% |
| 44 | *Eremothecium* | 0.00% | 0.00% | 0.10% | 0.00% |
| 45 | *Hazslinszkyomyces* | 0.00% | 0.00% | 0.00% | 0.10% |
| 46 | *Nothophoma* | 0.00% | 0.80% | 0.00% | 0.00% |
| 47 | *Exophiala* | 0.00% | 0.00% | 0.10% | 0.00% |
| 48 | *Westerdykella* | 0.00% | 0.00% | 0.10% | 0.00% |
| 49 | *Rhodotorula* | 0.00% | 0.00% | 0.10% | 0.00% |
| 50 | *Sordaria* | 0.00% | 0.10% | 0.10% | 0.00% |
| 51 | *Clitopilus* | 0.00% | 0.00% | 0.10% | 0.00% |
| 52 | *Neophysalospora* | 0.00% | 0.00% | 0.10% | 0.00% |
| 53 | *Pseudopithomyces* | 0.00% | 0.00% | 0.00% | 0.10% |
| 54 | *Ciliophora* | 0.00% | 0.00% | 0.10% | 0.00% |
| 55 | *Rhamphoria* | 0.00% | 0.00% | 0.10% | 0.00% |
| 56 | *Pseudocoleophoma* | 0.00% | 0.10% | 0.00% | 0.00% |
| 57 | *Buckleyzyma* | 0.00% | 0.00% | 0.00% | 0.00% |
| 58 | *Paracamarosporium* | 0.00% | 0.00% | 0.10% | 0.00% |
| 59 | *Paraophiobolus* | 0.00% | 0.00% | 0.10% | 0.00% |
| 60 | *Remotididymella* | 0.00% | 0.20% | 0.00% | 0.00% |
| 61 | *Arthrinium* | 0.00% | 0.00% | 0.10% | 0.00% |
| 62 | *Preussia* | 0.00% | 0.00% | 0.00% | 0.00% |
| 63 | *Hyphozyma* | 0.00% | 0.00% | 0.10% | 0.00% |
| 64 | *Moesziomyces* | 0.00% | 0.00% | 0.00% | 0.00% |
| 65 | *Entyloma* | 0.00% | 0.00% | 0.00% | 0.10% |
| 66 | *Millerozyma* | 0.00% | 0.00% | 0.00% | 0.00% |
| 67 | *Graphiola* | 0.00% | 0.00% | 0.10% | 0.00% |
| 68 | *Kwoniella* | 0.00% | 0.00% | 0.10% | 0.00% |
| 69 | *Pleiochaeta* | 0.00% | 0.00% | 0.10% | 0.00% |
| 70 | *Neophaeococcomyces* | 0.00% | 0.00% | 0.00% | 0.00% |
| 71 | *Microdochium* | 0.00% | 0.00% | 0.00% | 0.00% |
| 72 | *Vishniacozyma* | 0.00% | 0.00% | 0.00% | 0.00% |
| 73 | *Papiliotrema* | 0.00% | 0.00% | 0.00% | 0.00% |
| 74 | *Dictyosporium* | 0.00% | 0.00% | 0.00% | 0.00% |
| 75 | *Resinicium* | 0.00% | 0.00% | 0.00% | 0.00% |
| 76 | *Unguiculariopsis* | 0.00% | 0.00% | 0.00% | 0.00% |
| 77 | *Parapyrenochaeta* | 0.00% | 0.00% | 0.00% | 0.00% |
| 78 | *Magnibotryascoma* | 0.00% | 0.00% | 0.00% | 0.00% |
| 79 | *Schizopora* | 0.00% | 0.00% | 0.00% | 0.00% |
| 80 | *Phaeosphaeria* | 0.00% | 0.00% | 0.00% | 0.00% |
| 81 | *Arcticomyces* | 0.00% | 0.20% | 0.00% | 0.00% |
| 82 | *Cryptococcus* | 0.00% | 0.20% | 0.00% | 0.00% |
| 83 | *Auriculoscypha* | 0.00% | 0.20% | 0.00% | 0.00% |
| 84 | *Sydowia* | 0.00% | 0.00% | 0.00% | 0.00% |
| 85 | *Candida* | 0.00% | 0.00% | 0.00% | 0.00% |
| 86 | *Pseudozyma* | 0.00% | 0.00% | 0.00% | 0.00% |
| 87 | *Saitozyma* | 0.00% | 0.00% | 0.00% | 0.00% |
| 88 | *Robillarda* | 0.00% | 0.00% | 0.00% | 0.00% |
| 89 | *Apseudocercosporella* | 0.00% | 0.20% | 0.00% | 0.00% |
| 90 | *Knufia* | 0.00% | 0.00% | 0.00% | 0.00% |
| 91 | *Pilobolus* | 0.00% | 0.00% | 0.00% | 0.00% |
| 92 | *Cutaneotrichosporon* | 0.00% | 0.00% | 0.00% | 0.00% |
|  | Total | 100.00% | 100.00% | 100.00% | 100.00% |

**S. table 3**

Identified rumen metabolite compounds

|  | Compound Name | CAS | Retention Time | Chemical family |
| --- | --- | --- | --- | --- |
| 1 | Trifluoroacetyl-lavandulol | 58461-27-1 | 20.8942 | Alcohol |
| 2 | Phenyl ethyl alcohol | 000060-12-8 | 14.4856 | Alcohol |
| 3 | Terpinen-4-ol | 000100-49-2 | 20.2671 | Alcohol |
| 4 | 1-Hexyn-3-ol | 000105-31-7 | 14.413 | Alcohol |
| 5 | Junenol | 000472-07-1. | 20.2449 | Alcohol |
| 6 | Terpinen-4-ol | 000562-74-3 | 14.8027 | alcohol |
| 7 | α-Benzenemethanol | 006589-55-5 | 2.7528 | Alcohol |
| 8 | 3-Methoxybenzyl alcohol | 006971-51-3 | 11.4652 | alcohol |
| 9 | 3-methyl,1-Hexanol | 013231-81-7 | 8.0365 | alcohol |
| 10 | Benzenemethanol | 048115-38-4 | 2.3272 | alcohol |
| 11 | anine ethylamide | 001999-43-5 | 20.4015 | Amide |
| 12 | Urea | 000057-13-6 | 3.2582 | Amine |
| 13 | 2-Pyrimidinamine | 000109-12-6 | 11.0175 | Amine |
| 14 | Amphetamine | 000300-62-9 | 18.2966 | Amine |
| 15 | 1-Anthracenamine | 000610-49-1 | 11.0398 | Amine |
| 16 | 2-Ethoxyamphetamine | 135014-84-5 | 1.9687 | Amine |
| 17 | Ethoxyamphetamine | 135014-84-5 | 3.7607 | Amine |
| 18 | p-Cymene | 000099-87-6 | 12.227 | Aromatic Hydrocarbon |
| 19 | Ethylbenzene | 000100-41-4 | 15.0261 | Aromatic Hydrocarbon |
| 20 | Toluene | 000108-88-3 | 5.3507 | Aromatic Hydrocarbon |
| 21 | o-Cymene | 000527-84-4 | 12.2268 | Aromatic Hydrocarbon |
| 22 | Benzene, 1-2-methyl | 000611-14-3 | 21.2975 | Aromatic Hydrocarbon |
| 23 | Naphthalene, decahydro-1,4a-dimethyl | 001008-18-0 | 19.1027 | Aromatic Hydrocarbon |
| 24 | 1-Benzopyran-2-one, 6-hydroxy-7-methoxy-4-methyl | 006345-62-6 | 21.2978 | Aromatic Hydrocarbon |
| 25 | 1-(1,1-dimethylethoxy)-2-methyl,Benzene | 015359-98-5 | 2.6837 | Aromatic Hydrocarbon |
| 26 | Benzene, 1-(1,1-dimethylethoxy)-2-methyl | 015359-98-5 | 12.7867 | Aromatic Hydrocarbon |
| 27 | 1-Bromo-2-benzyloxybenzene | 031575-75-4 | 12.2266 | Aromatic Hydrocarbon |
| 28 | Heptanoic acid | 000111-14-8 | 16.7061 | carboxylic acid |
| 29 | Hydrocinnamic acid | 000501-52-0 | 15.3623 | carboxylic acid |
| 30 | Octadecanoic acid | 000057-11-4 | 25.889 | carboxyllic acid |
| 31 | Pentanoic acid | 000097-61-0 | 9.5168 | Carboxyllic acid |
| 32 | Cyclohexanecarboxylic acid | 000098-89-5 | 17.5572 | Carboxyllic acid |
| 33 | Butyl butanoate | 000109-21-7 | 17.0643 | Ester |
| 34 | 3-Hydroxymandelic acid, ethyl ester | 017066-67-0 | 14.2424 | Ester |
| 35 | Pentanoic acid, 1,1-dimethylpropyl ester | 023361-78-6 | 19.9983 | Ester |
| 36 | 3-Hydroxymandelic acid, ethyl ester | 1000071-88-9 | 13.1896 | Ester |
| 37 | Dodecanoic acid, 2-hexen-1-yl ester | 1000159-97-0 | 12.5626 | ester |
| 38 | Carbamic acid, benzyl ester | 1000314-73-3 | 20.581 | ester |
| 39 | Glutaric acid, dodecyl tetrahydrofurfuryl ester | 1000359-66-8 | 12.988 | Ester |
| 40 | Isoamyl benzyl ether | 000122-73-6 | 6.0899 | Ether |
| 41 | Ethyl allophanate | 000626-36-8 | 1.9688 | Ether |
| 42 | 1-Methyl-cyclohexyl propionate | 091328-37-9 | 12.9882 | ether |
| 43 | Carbon dioxide | 000124-38-9 | 1.5657 | Greenhouse gas |
| 44 | Cyclopentene-3,5-dimethylene | 000000-00-0 | 11.6667 | Hydrocarbon |
| 45 | Nonane | 000111-84-2 | 9.6734 | hydrocarbon |
| 46 | Dodecane | 000112-40-3 | 14.5336 | Hydrocarbon |
| 47 | Eicosane | 000112-95-8 | 22.6865 | Hydrocarbon |
| 48 | Tricyclene | 000508-32-7 | 9.4942 | Hydrocarbon |
| 49 | Hexadecane | 000544-76-3 | 19.8193 | Hydrocarbon |
| 50 | Methyl cyclohexene | 000591-47-9 | 12.4507 | Hydrocarbon |
| 51 | 4-methyl-Cyclohexene | 000591-47-9 | 11.1292 | Hydrocarbon |
| 52 | Cyclohexene, 4-methyl | 000591-47-9 | 11.7118 | Hydrocarbon |
| 53 | 1,3-Cyclohexadiene | 000592-57-4 | 11.6446 | Hydrocarbon |
| 54 | Cyclopentanedione-3-methyl | 000765-70-8 | 11.6666 | Hydrocarbon |
| 55 | 2,6-Dimethyl-1,3,6-heptatriene | 000928-67-6 | 14.108 | hydrocarbon |
| 56 | Undecane | 001120-21-4 | 11.3754 | Hydrocarbon |
| 57 | 3-methyl-Cyclopentene | 001120-62-3 | 12.0249 | Hydrocarbon |
| 58 | Cyclohexane, 1,2,3-trimethyl | 001678-97-3 | 9.203 | Hydrocarbon |
| 59 | cis-2,6-Dimethyl-2,6-octadiene | 002492-22-0 | 10.9276 | Hydrocarbon |
| 60 | 1-Methylcyclohexa-2,4-diene | 004313-57-9 | 11.1292 | Hydrocarbon |
| 61 | 2,4-Heptadiene, 2,6-dimethyl | 004634-87-1 | 7.7476 | Hydrocarbon |
| 62 | 2-Nonene | 006434-78-2 | 9.0239 | Hydrocarbon |
| 63 | 2-Octene, 3,7-dimethyl | 006874-32-4 | 11.1295 | Hydrocarbon |
| 64 | 2-Octene, 3,7-dimethyl | 006874-32-4 | 11.1294 | Hydrocarbon |
| 65 | 3-Octene | 007642-04-8 | 6.4034 | Hydrocarbon |
| 66 | 2- Octene | 013389-42-9 | 7.3441 | hydrocarbon |
| 67 | 4-Octene | 014850-23-8 | 6.4033 | Hydrocarbon |
| 68 | 2,6-dimethyl-Undecane | 017301-23-4 | 14.7351 | hydrocarbon |
| 69 | Undecane, 2,6-dimethyl | 017301-23-4 | 6.4033 | Hydrocarbon |
| 70 | 3,6-dimethyl-Decane | 017312-53-7 | 12.7868 | hydrocarbon |
| 71 | Neoclovene (II), dihydro | 030824-81-8 | 19.2373 | Hydrocarbon |
| 72 | 1,5-Heptadiene, 2,3,6-trimethyl- | 033501-88-1 | 11.5102 | Hydrocarbon |
| 73 | 3,7-Dimethyl-3-octyl methylphosphonofluoridate | 0345260-82-4 | 22.8205 | Hydrocarbon |
| 74 | 3,3,5,5-Tetramethylcyclopentene | 038667-10-6 | 12.0254 | hydrocarbon |
| 75 | 3-Heptyne, 5-ethyl-5-methyl | 061228-10-2 | 11.3756 | Hydrocarbon |
| 76 | 1,5,9-Undecatriene, 2,6,10-trimethyl | 062951-96-6 | 17.49 | Hydrocarbon |
| 77 | 4-Octene, 2,6-dimethyl | 062960-77-4 | 9.9425 | hydrocarbon |
| 78 | Dauca-5,8-diene | 142928-08-3 | 19.5733 | Hydrocarbon |
| 79 | 2,3-Dimethyl-1-hexene | 16746-86-4 | 14.7352 | Hydrocarbon |
| 80 | 1,1'-Dianthrimide | 000082-22-4 | 16.863 | Imide |
| 81 | 3-methyl-Indole | 000083-34-1 | 17.4453 | Indole |
| 82 | 1H-Indole, 3-methyl- | 000083-34-1 | 17.8708 | indole |
| 83 | Skatole | 000083-34-1 | 17.3108 | Indole |
| 84 | Indole | 000120-72-9 | 16.0117 | Indole |
| 85 | Indole, 6-methyl | 003420-02-8 | 18.2741 | Indole |
| 86 | Indole, 1-methyl-2-phenyl | 003558-24-5 | 13.1003 | indole |
| 87 | 3-Octanone | 000106-68-3 | 11.5326 | Ketone |
| 88 | 6-methyl-5-Hepten-2-one | 000110-93-0 | 11.5098 | Ketone |
| 89 | 2-Undecanone | 000112-12-9 | 16.4824 | Ketone |
| 90 | 2-Decanone | 000693-54-9 | 13.9961 | ketone |
| 91 | 2- Nonanone | 000821-55-6 | 12.921 | ketone |
| 92 | Cyclohexanone | 092368-82-6 | 19.9291 | Ketone |
| 93 | Camphor | 000076-22-2 | 13.7721 | Monoterpene |
| 94 | Camphene | 000079-92-5 | 10.6816 | Monoterpene |
| 95 | α-Pinene | 000080-56-8 | 10.3904 | Monoterpene |
| 96 | Terpinene | 000099-85-4 | 10.1268 | Monoterpene |
| 97 | β -Pinene | 000127-91-3 | 10.7036 | Monoterpene |
| 98 | Limonene | 000138-86-3 | 12.2718 | Monoterpene |
| 99 | 1-p-Menthene | 000500-00-5 | 11.7115 | Monoterpene |
| 100 | 3-p-Menthene | 000500-00-5 | 10.8381 | Monoterpene |
| 101 | 3-Carene | 000554-61-0 | 12.7417 | Monoterpene |
| 102 | Terpinolene | 000586-62-9 | 9.5165 | Monoterpene |
| 103 | Citronellene | 002436-90-0 | 10.6714 | Monoterpene |
| 104 | α-Thujene | 002867-05-2 | 9.2029 | Monoterpene |
| 105 | β -Terpinene | 005392-40-5 | 12.7866 | Monoterpene |
| 106 | Delta -2 Carene | 013466-78-9 | 12.4766 | Monoterpene |
| 107 | delta-Carene | 013466-78-9 | 19.3905 | Monoterpene |
| 108 | α-Cubebene | 017699-14-8 | 17.3111 | Monoterpene |
| 109 | β -cis-Bergamotene | 018252-46-5 | 17.8931 | Monoterpene |
| 110 | β -Citronellene | 10281-55-7 | 11.2381 | Monoterpene |
| 111 | Geranyl linalool | 68931-30-6 | 10.3227 | Monoterpene |
| 112 | Pyrrole, 1-methyl | 000096-54-8 | 11.3082 | Others |
| 113 | Dibenzo carbazole | 000207-84-1 | 13.7275 | Others |
| 114 | 2-Mercapto-4-phenylthiazole | 002103-88-0 | 11.0398 | Others |
| 115 | 2-Heptylfuran | 003777-71-7 | 17.445 | Others |
| 116 | p-Cresol | 000106-44-5 | 13.1677 | Phenolic |
| 117 | 4-ethyl,Phenol | 000123-07-9 | 14.7578 | Phenolic |
| 118 | Phenol, 2,4-dimethyl- | 000105-67-9 | 15.17 | phenollic |
| 119 | Phenol, 3-methyl- | 000108-39-4 | 15.4743 | phenollic |
| 120 | Phenol | 000108-95-2 | 11.6222 | phenollic |
| 121 | Phenol, 3-propyl- | 000621-27-2 | 16.348 | Phenollic |
| 122 | 2-propyl-Phenol | 000644-35-9 | 15.4966 | phenollic |
| 123 | Selina-3,7(11)-diene | 006813-21-4 | 18.7217 | Sesquiterpene |
| 124 | β- Caryophyllene | 000087-44-5 | 18.0949 | Sesquiterpene |
| 125 | α -Guaiene | 000088-84-6 | 19.7524 | Sesquiterpene |
| 126 | α -Selinene | 000473-13-2 | 19.5282 | Sesquiterpene |
| 127 | β -Selinene | 000473-13-2 | 19.5281 | Sesquiterpene |
| 128 | Cadinene | 000483-76-1 | 19.4613 | Sesquiterpene |
| 129 | Patchoulene | 000508-55-4 | 19.3492 | Sesquiterpene |
| 130 | α-Copaene | 003856-25-5 | 17.6695 | sesquiterpene |
| 131 | Valencene | 004630-07-3 | 18.6771 | Sesquiterpene |
| 132 | β -Dihydro agarofuran | 005956-09-2 | 19.1715 | Sesquiterpene |
| 133 | β -Amorphene | 006980-46-7 | 19.5282 | Sesquiterpene |
| 134 | α-Cubebene | 013744-15-5 | 18.9236 | Sesquiterpene |
| 135 | Sibirene | 014029-18-6 | 19.73 | Sesquiterpene |
| 136 | α-Gurjunene | 017334-55-3 | 17.5797 | sesquiterpene |
| 137 | β -Gurjunene | 017334-55-3 | 18.453 | Sesquiterpene |
| 138 | cis-Cadina-1(6),4-diene | 020085-11-4 | 19.2144 | Sesquiterpene |
| 139 | β -Elemene | 020307-84-0 | 17.1095 | Sesquiterpene |
| 140 | β -Muurolene | 024268-39-1 | 18.9684 | Sesquiterpene |
| 141 | -Cuprenene | 029621-78-1 | 21.3199 | Sesquiterpene |
| 142 | Cis-Calamenene | 072937-55-4 | 19.5731 | sesquiterpene |
| 143 | t-Cadina-1,4-diene | 246522-85-0 | 21.8349 | Sesquiterpene |
| 144 | Amorpha-4,7(11)-diene | 486998-53-2 | 20.4463 | Sesquiterpene |
| 145 | Italicene | 94535-52-1 | 20.0657 | Sesquiterpene |
| 146 | Dimethyl sulfide | 000075-18-3 | 2.0134 | thiol |
| 147 | Disulfide, dimethyl | 000624-92-0 | 4.6788 | Thiol |
| 148 | 1-Propene-1-thiol | 000870-23-5 | 21.6109 | Thiol |
| 149 | Cyclic octaatomic sulfur | 010544-50-0 | 25.4188 | Thiol |
| 150 | Mint sulfide | 072445-42-2 | 21.611 | Thiol |
| 151 | Acetic acid | 000064-19-7 | 4.0967 | Volatile fatty acid |
| 152 | Propanoic acid | 000079-09-4 | 6.3147 | Volatile fatty acid |
| 153 | 2-methyl,Propanoic acid | 000079-31-2 | 7.2382 | Volatile fatty acid |
| 154 | Valeric acid-3-methyl | 000105-43-1 | 9.2929 | Volatile fatty acid |
| 155 | Butanoic acid | 000107-92-6 | 7.68 | Volatile fatty acid |
| 156 | 2-methyl,Butanoic acid | 000116-53-0 | 9.6512 | Volatile fatty acid |
| 157 | Butanoic acid-2-methyl | 000116-53-0 | 9.1586 | Volatile fatty acid |
| 158 | 2-methyl, Butanoic acid | 000116-53-0 | 9.5839 | Volatile fatty acid |
| 159 | Hexanoic acid | 000142-62-1 | 10.1214 | Volatile fatty acid |
| 160 | 3-methyl-Butanoic acid | 000503-74-2 | 8.7325 | Volatile fatty acid |
| 161 | Isovaleric acid | 000503-74-2 | 9.36 | Volatile fatty acid |
| 162 | Butanoic acid, 3-methyl | 000503-74-2 | 9.5168 | Volatile fatty acid |

**S. table 4**

List of primers used for plant diet assessment

|  |  | Plant diet assessment primers | | |
| --- | --- | --- | --- | --- |
| Primer name | Binding site | Sequence (50–30 ) | Target gene | Primer Tm |
| trnH-psbA | Forward | CGCGCATGGTGGATTCACAATCC | Intergenic | 66.4^o^C 55.5 ^o^C |
|  | Reverse | GTTATGCATGAACGTAATGCT | spacer region | |
| rbcL2 | Forward | TATGTAGCTTAYCCMTTAGACCTTTTTGAAGA | Chloroplast | 66.1^o^C 68.7^o^C |
|  | Reverse | GCTTCGGCACAAAAKARGAARCGGTCTC | | |
| rbcL-A | Forward | ATGTCACCACAAACAGAGACTAAAGC | Chloroplast | 64.7^o^C 56.4^o^C |
|  | Reverse | GTAAAATCAAGTCCACCRCG | | |
